# Association between high-sensitivity troponin and mortality risk in individuals with early kidney disease: A population-based cohort study

**DOI:** 10.1097/MD.0000000000043014

**Published:** 2025-06-27

**Authors:** Ming-Yan Jiang, Wen-Shiann Wu

**Affiliations:** a Department of Internal Medicine, Renal Division, Chi Mei Medical Center, Tainan, Taiwan; b Department of Pharmacy, Chia Nan University of Pharmacy & Science, Tainan, Taiwan; c Department of Internal Medicine, Cardiovascular Division, Chi Mei Medical Center, Tainan, Taiwan.

**Keywords:** albuminuria, cardiovascular mortality, early kidney disease, high-sensitivity troponin (hs-Tn), subclinical myocardial injury

## Abstract

Cardiac troponins are prognostic biomarkers for cardiovascular disease (CVD) and mortality, but their significance in individuals with early kidney disease, defined as preserved estimated glomerular filtration rate but albuminuria, remains unclear. This study evaluated the association between high-sensitivity troponin I (hs-TnI) and troponin T (hs-TnT) with all-cause and cardiovascular mortality in this population. This retrospective cohort study used data from the 1999 to 2004 National Health and Nutrition Examination Survey. We included 1296 adults (≥18 years) with estimated glomerular filtration rate ≥ 60 mL/min/1.73 m² and urinary albumin-creatinine ratio ≥ 30 mg/g. Serum hs-TnI and hs-TnT levels were categorized into quintiles. The primary outcomes were all-cause and cardiovascular mortality, determined via National Health and Nutrition Examination Survey-linked National Death Index data through December 31, 2019. The mean age of participants was 51.4 ± 0.7 years, and 45.9% were male. Over a median follow-up of 219 months, 601 participants died, including 185 from CVD. Higher hs-Tn levels were associated with older age, hypertension, diabetes, and greater urinary albumin–creatinine ratio. Each one-quintile increase in hs-TnI was associated with a 26% higher risk of all-cause mortality (hazard ratios [HR]: 1.26; 95% CI: 1.13–1.41; *P*-trend < .001) and a 33% higher risk of CVD mortality (HR: 1.33; 95% CI: 1.04–1.71; *P*-trend < .05). Similar associations were observed for hs-TnT (HR: 1.56 for all-cause mortality; 95% CI: 1.37–1.77; *P* < .001; HR: 1.64 for CVD mortality; 95% CI: 1.33–2.03; *P* < .001). Sensitivity analyses excluding individuals with preexisting CVD and early deaths confirmed the robustness of these findings. Elevated hs-TnI and hs-TnT independently predict all-cause and cardiovascular mortality in individuals with early kidney disease. These findings highlight the potential role of hs-Tn measurement in enhancing cardiovascular risk stratification. Incorporating hs-Tn testing into routine assessments may help identify individuals who could benefit from earlier preventive interventions to reduce long-term cardiovascular risk; however, further research is needed to validate its clinical utility.

## 1. Introduction

Chronic kidney disease (CKD) and cardiovascular disease (CVD) frequently coexist with metabolic conditions such as diabetes mellitus, forming the basis of the emerging cardio-kidney-metabolic syndrome.^[1]^ This constellation confers a markedly higher risk of adverse outcomes than any individual condition alone.^[1]^ Albuminuria, a hallmark of early kidney damage, is also a strong independent predictor of both cardiovascular events and mortality, even in individuals with preserved kidney function.^[2]^ Among patients with established CVD, increasing levels of albuminuria are closely linked to a greater burden of both cardiovascular and renal complications.^[3,4]^ As a marker of systemic endothelial dysfunction, heightened cardiovascular stress, and vascular injury,^[1,5]^ albuminuria underscores the biologic and clinical interplay between kidney dysfunction and myocardial injury.

High-sensitivity cardiac troponin (hs-Tn) is a well-established biomarker of myocardial injury and is widely used in diagnosis of acute coronary syndromes. Beyond its diagnostic role, hs-Tn is also a prognostic marker for cardiovascular and all-cause mortality across diverse populations, including the general population,^[6]^ individuals with or without diabetes,^[7,8]^ and those with established CVD.^[9]^ Additionally, previous studies have demonstrated that elevated troponin levels are predictive of mortality in both dialysis-dependent and non-dialysis-dependent advanced CKD populations.^[10–12]^ However, while high-sensitivity troponin I (hs-TnI) and high-sensitivity troponin T (hs-TnT) assays have improved the sensitivity for detecting myocardial injury, their interpretation in patients with kidney impairment remains challenging due to chronically elevated troponin levels that may not necessarily reflect acute ischemia.^[13,14]^

Chronic kidney disease affects more than 10% of the global population, with early-stage CKD accounting for over 7%.^[15,16]^ However, this prevalence is likely underestimated due to the lack of widespread screening and early detection programs, leading to substantial unawareness of early kidney dysfunction.^[17]^ Although hs-Tn is a recognized prognostic marker in advanced CKD, its significance in individuals with early kidney disease, defined as preserved estimated glomerular filtration rate (eGFR) but albuminuria, remains unclear. Traditional cardiovascular risk markers often fail to detect early subclinical myocardial injury, especially for those with early kidney disease.^[18]^ High-sensitivity troponins may offer additional prognostic insight, but their role in risk prediction among individuals with early CKD is underexplored. Given the well-documented association between albuminuria and cardiovascular risk,^[19]^ this study aims to evaluate the relationship between hs-Tn levels and long-term mortality risk in individuals with preserved eGFR but albuminuria, using nationally representative data.

## 2. Methods

### 2.1. Data source and study population

This study utilized data from the National Health and Nutrition Examination Survey (NHANES), a nationally representative cross-sectional survey conducted by the National Center for Health Statistics (NCHS). NHANES employs multistage probability sampling to assess the health and nutritional status of the noninstitutionalized U.S. population through structured interviews, physical examinations, and laboratory testing. The NHANES dataset provides detailed biomarker and clinical information and can be linked to survival status and cause of death, making it well-suited for evaluating long-term risk predictors in the U.S. population. For this analysis, we included data from the 1999 to 2000 to 2003 to 2004 survey cycles, which were the only cycles with available hs-Tn measurements. NHANES data are publicly available through the NCHS website (https://wwwn.cdc.gov/nchs/nhanes/Default.aspx). The study protocols were approved by the NCHS Research Ethics Review Board, and all participants provided written informed consent in accordance with the Declaration of Helsinki.

The initial sample included 17,061 adults aged ≥ 18 years. We included 1393 participants with preserved kidney function (eGFR ≥ 60 mL/min/1.73 m²) and albuminuria (urinary albumin-creatinine ratio [ACR] ≥ 30 mg/g). After excluding 97 individuals missing hs-TnI and/or hs-TnT measurements, the final analytical sample consisted of 1296 participants.

### 2.2. Exposure variables

High-sensitivity troponin was measured from stored serum specimens (stored at −80 °C prior to analysis), with testing performed between 2018 and 2020 at the University of Maryland School of Medicine, Baltimore, MD. Hs-TnI was measured using the Abbott ARCHITECT i2000SR assay, which has a limit of detection (LoD) of 1.7 ng/L. The coefficients of variation (CVs) were 6.4% for concentrations between 8 to 16 ng/L, 3.5% for 169 to 314 ng/L, and 6.7% for 2758 to 4444 ng/L. Hs-TnT was measured using the Roche Cobas e601 assay with generation 5 Elecsys reagents. The LoD for this assay is 3 ng/L, with CVs of 3.1% for concentrations between 26 to 31 ng/L and 2.0% for 2005 to 2216 ng/L. Participants were categorized into quintiles based on their hs-TnI or hs-TnT concentrations for analysis.

### 2.3. Outcome variables

Mortality status was determined by linking NHANES data to the National Death Index using probabilistic matching and death certificate review. The International Classification of Diseases–Tenth Revision (ICD-10) codes were used to define cause-specific mortality. Deaths from CVD were identified using ICD-10 codes I00 to I09, I11, I13, and I20 to I51, as included in the publicly available NHANES-linked mortality file. Follow-up began at the baseline NHANES interview and continued until death or the study endpoint (December 31, 2019), whichever occurred first.

### 2.4. Covariates

Several demographic, socioeconomic, lifestyle, clinical, and laboratory variables were included as covariates. Demographic factors included age, sex, and self-reported race/ethnicity (non-Hispanic White, non-Hispanic Black, Hispanic, or other/multiracial). Socioeconomic factors included education level (≤high school vs some college or higher), marital status (married/living with a partner vs single/widowed/divorced/separated), and family income-to-poverty ratio, calculated as total family income divided by the federal poverty threshold, adjusted for family size, year, and state. Lifestyle factors included smoking status (never, former, or current smoker). Clinical variables included body mass index (BMI), waist circumference, systolic and diastolic blood pressure (averaged from up to 4 measurements), and the presence of diabetes mellitus, hypertension, or CVD.

Diabetes was defined as self-reported diagnosis or current use of diabetes medications, or hemoglobin A1c ≥ 6.5%. Hypertension was defined as self-reported diagnosis or antihypertensive medication use. CVD was defined as a self-reported history of congestive heart failure, coronary heart disease, angina, myocardial infarction, or stroke. Laboratory measurements included serum creatinine, eGFR, urinary albumin-to-creatinine ratio (UACR), and hemoglobin. The eGFR was calculated using the 2021 Chronic Kidney Disease Epidemiology Collaboration (CKD-EPI) equation.

### 2.5. Sensitivity analyses

Several sensitivity analyses were conducted to ensure the robustness of our findings. First, we examined the predictive significance of hs-TnI measured using 2 additional commercial assays: the Siemens Centaur XP and the Ortho Vitros 3600. Hs-TnI measured using the Siemens Centaur XPT (TNIH assay) had a LoD of 1.6 ng/L. The CVs were 3.8% at concentrations of 12 to 28 ng/L and 2.6% at concentrations of 9000 to 21,000 ng/L. Hs-TnI measured using the Ortho Vitros 3600 platform had an LoD of 0.39 ng/L. The CVs were 4.2% at concentrations of 6 to 16 ng/L and 2.8% at concentrations of 17,511 to 21,403 ng/L.

Second, to evaluate the potential impact of preexisting CVD on our results, we repeated our analyses after excluding individuals with self-reported CVD. Third, to address potential reverse causation, we conducted additional analyses excluding participants who died within the first 2 years of follow-up.

### 2.6. Statistical analysis

All statistical analyses accounted for the complex survey design of NHANES by applying sample weights to adjust for nonresponse, oversampling, and noncoverage, ensuring representativeness of the U.S. population. Continuous variables were expressed as survey-weighted means with standard errors, and categorical variables were reported as counts with survey-weighted proportions.

To explore predictors and associations, weighted linear regression was used to analyze factors associated with log-transformed hs-TnI levels. Weighted logistic regression was performed to identify potential modifiable risk factors for being in the highest quintile of hs-TnI and hs-TnT, adjusting for age, sex, and race/ethnicity. Results were reported as odds ratios (OR) with 95% confidence intervals (CI).

Survival analysis was conducted using Kaplan–Meier curves and log-rank tests to compare mortality differences across hs-TnI and hs-TnT quintiles. Additionally, weighted Cox proportional hazards regression models were used to estimate hazard ratios (HR) with 95% CIs for all-cause and cardiovascular mortality, adjusting for age, sex, race/ethnicity, BMI, diabetes, hypertension, CVD, smoking status, family income-to-poverty ratio, hemoglobin, UACR, and eGFR. All statistical analyses were performed using SAS version 9.4 (SAS Institute, Cary, NC, USA).

## 3. Results

The mean age of participants was 51.4 ± 0.7 years, with 45.9% being male. The racial/ethnic distribution included 65.5% White, 13.0% Black, and 16.3% Hispanic (Table 1). Higher hs-TnI levels were significantly associated with older age, male sex, Black race, lower income-poverty ratio, greater waist circumference, and a higher prevalence of hypertension and CVD (Table S1, Supplemental Digital Content, https://links.lww.com/MD/P250).

**Table 1:** Baseline characteristics of the study population by quintiles of high-sensitivity troponin I (hs-TnI) levels.

	Total	Quintile 1	Quintile 2	Quintile 3	Quintile 4	Quintile 5
hs-TnI range (ng/L)	0–862.8	0–1.2	1.3–2.1	2.2–3.4	3.5–6.5	6.6–862.8
No. of participants	1296*	256	250	263	257	261
Sociodemographic						
Age	51.4 ± 0.7	33.5 ± 0.9	46.0 ± 1.0	56.6 ± 1.2	63.6 ± 1.3	63.6 ± 1.1
Sex						
Male	620 (45.9)	49 (21.5)	102 (42.0)	152 (56.6)	140 (52.0)	173 (65.2)
Female	676 (54.1)	207 (78.5)	148 (58.0)	111 (43.4)	117 (48.0)	88 (34.8)
Race						
White	570 (65.5)	93 (57.5)	110 (70.6)	124 (66.3)	119 (68.2)	120 (64.7)
Black	260 (13.0)	48 (10.7)	41 (10.7)	50 (11.1)	56 (15.2)	64 (19.7)
Hispanic	429 (16.3)	105 (24.6)	93 (14.9)	77 (15.4)	75 (11.2)	75 (13.2)
Others	37 (5.3)	10 (7.2)	6 (3.8)	12 (7.2)	7 (5.3)	2 (2.4)
Marital status						
Married	702 (57.1)	113 (50.6)	143 (63.1)	157 (61.6)	134 (54.0)	149 (55.8)
Single	566 (42.9)	141 (49.4)	102 (36.9)	99 (38.4)	116 (46.0)	105 (44.2)
Educational level						
≤ High school	777 (55.6)	120 (49.0)	138 (54.0)	160 (52.5)	175 (67.0)	179 (58.4)
≥ College	423 (44.4)	75 (51.0)	91 (46.0)	93 (47.5)	79 (33.0)	81 (41.6)
Family income-to-poverty ratio	2.55 ± 0.10	2.37 ± 0.15	2.71 ± 0.13	2.73 ± 0.17	2.58 ± 0.17	2.28 ± 0.13
Measurement						
Body mass index (kg/m^2^)	29.2 ± 0.4	26.5 ± 0.6	29.8 ± 0.7	30.2 ± 0.6	29.8 ± 0.5	30.5 ± 0.8
Waist circumference (cm)	100.3 ± 0.9	89.7 ± 1.5	101.0 ± 1.8	103.5 ± 1.5	104.7 ± 1.2	105.8 ± 1.9
Systolic blood pressure (mm Hg)	134.1 ± 0.9	114.3 ± 1.1	126.7 ± 1.2	139.4 ± 1.8	148.9 ± 1.6	148.5 ± 2.1
Diastolic blood pressure (mm Hg)	74.6 ± 0.6	71.6 ± 0.9	73.5 ± 1.0	75.2 ± 1.1	77.2 ± 1.6	76.7 ± 1.1
Health behavior						
Smoking status						
Never	549 (44.8)	110 (51.4)	117 (44.5)	113 (44.0)	106 (44.4)	99 (37.9)
Former	365 (27.6)	27 (16.7)	54 (22.1)	80 (29.8)	101 (32.4)	101 (40.7)
Current	285 (27.7)	58 (32.0)	57 (33.3)	60 (26.2)	47 (23.3)	60 (21.4)
Comorbidity						
Diabetes	387 (25.3)	37 (11.2)	75 (24.9)	85 (26.5)	90 (34.2)	97 (33.9)
Hypertension	573 (41.4)	45 (17.7)	74 (23.6)	132 (52.6)	158 (60.8)	159 (64.1)
Cardiovascular disease	180 (11.5)	2 (0.5)	8 (3.1)	27 (9.2)	60 (22.5)	82 (28.8)
Laboratory data						
Hemoglobin (g/dL)	14.4 ± 0.1	14.0 ± 0.1	14.6 ± 0.1	14.7 ± 0.1	14.4 ± 0.1	14.5 ± 0.1
UACR (mg/g)	156.7 ± 12.6	94.4 ± 10.1	118.2 ± 15.5	140.3 ± 22.7	191.7 ± 36.7	266.2 ± 47.8
eGFR (mL/min/1.73 m²)	99.0 ± 0.8	114.6 ± 2.0	103.2 ± 1.4	94.9 ± 1.3	89.1 ± 1.2	88.1 ± 1.9
Hs-Troponin T (ng/L)	9.87 ± 0.32	3.98 ± 0.17	6.28 ± 0.46	8.24 ± 0.30	13.44 ± 0.84	21.44 ± 1.68

Continuous variables presented as mean ± standard error; categorical variables presented as counts (weighted proportion).

eGFR = estimated glomerular filtration rate, Hs-Troponin T = high-sensitivity Troponin T, UACR = urinary albumin-creatinine ratio.

* 9 participants missing high-sensitivity troponin I values.

In logistic regression analyses adjusted for age, sex, and race/ethnicity, participants in the highest quintile of hs-TnI were significantly more likely to have a lower income-poverty ratio, higher BMI, larger waist circumference, hypertension, and CVD (Table 2). Additionally, each 5 mm Hg increase in systolic blood pressure (SBP) was associated with a 9% higher likelihood of being in the highest hs-TnI quintile. Similarly, individuals in the highest hs-TnT quintile were more likely to have a lower income-poverty ratio, diabetes, hypertension, CVD, lower hemoglobin levels, and higher UACR (Table 2).

**Table 2:** Predictors of being in the highest quintile of high-sensitivity troponin I (hs-TnI) and troponin T (hs-TnT).

	Being in the highest quintile of hs-TnI		Being in the highest quintile of hs-TnT	
	Crude OR (95% CI)	Adjust OR (95% CI)#	Crude OR (95% CI)	Adjust OR (95% CI)#
Sociodemographic				
Age (every 10 years old)	1.58 (1.42–1.76)*	–	1.77 (1.58–1.97)*	–
Sex				
Male	2.54 (1.78–3.63)*	–	3.75 (2.54–5.54)*	–
Female	1		1	
Race/ethnicity				
White	1		1	1
Black	1.70 (1.19–2.44)**	–	0.93 (0.56–1.55)	–
Hispanic	0.80 (0.48–1.33)	–	0.39 (0.25–0.59)*	–
Others	0.41 (0.09–1.87)	–	0.36 (0.07–1.74)	–
Marital status				
Married	1	1		1
Single	1.07 (0.75–1.53)	1.34 (0.88–2.02)	1.00 (0.67–1.49)	1.46 (0.85–2.50)
Educational level				
≤High school	1	1	1	1
≥College	0.88 (0.59–1.30)	0.96 (0.65–1.43)	1.02 (0.72–1.45)	1.03 (0.67–1.58)
Family income-to-poverty ratio	0.88 (0.79–0.99)***	0.83 (0.74–0.94)**	0.90 (0.80–1.00)	0.79 (0.68–0.93)**
Measurement				
Body mass index (kg/m^2^)	1.03 (1.00–1.05)***	1.04 (1.01–1.08)***	1.01 (0.98–1.03)	1.02 (0.98–1.06)
Waist circumference (cm)	1.02 (1.01–1.03)*	1.02 (1.00–1.03)***	1.02 (1.00–1.03)**	1.01 (0.99–1.03)
Systolic blood pressure (5 mm Hg)	1.14 (1.10–1.17)*	1.09 (1.04–1.13)*	1.09 (1.05–1.13)*	1.02 (0.97–1.07)
Diastolic blood pressure (5 mm Hg)	1.07 (1.00–1.15)***	1.08 (1.00–1.16)	0.98 (0.91–1.05)	0.99 (0.92–1.07)
Health behavior				
Smoking status				
Never	1	1	1	1
Former	1.98 (1.30–3.01)**	1.25 (0.81–1.95)	2.14 (1.44–3.20)*	1.07 (0.64–1.78)
Current	0.90 (0.57–1.42)	1.04 (0.60–1.80)	0.82 (0.48–1.40)	0.90 (0.48–1.69)
Comorbidity				
Diabetes	1.65 (1.11–2.46)***	1.18 (0.75–1.86)	2.43 (1.79–3.31)*	1.95 (1.36–2.78)*
Hypertension	3.01 (2.02–4.48)*	1.99 (1.33–2.97)**	2.64 (1.81–3.85)*	1.77 (1.18–2.66)**
Cardiovascular disease	4.49 (2.86–7.05)*	2.52 (1.54–4.12)*	6.04 (4.01–9.10)*	2.96 (1.90–4.60)*
Laboratory data				
Hemoglobin (every 1 g/dL)	1.04 (0.93–1.15)	0.98 (0.84–1.15)	0.98 (0.87–1.11)	0.81 (0.70–0.94)**
UACR (every 10 mg/g)	1.01 (1.00–1.01)	1.01 (1.00–1.01)	1.01 (1.00–1.01)	1.00 (1.00–1.01)**
eGFR (every 10 mL/min/1.73 m²)	0.71 (0.62–0.81)*	0.92 (0.76–1.13)	0.65 (0.57–0.74)	0.86 (0.71–1.05)

eGFR = estimated glomerular filtration rate, UACR = urinary albumin-creatinine ratio.

† Adjusted for age, sex, and race/ethnicity.

* *P* < .001.

** *P* < .01.

*** *P* < .05.

**Figure 1.**
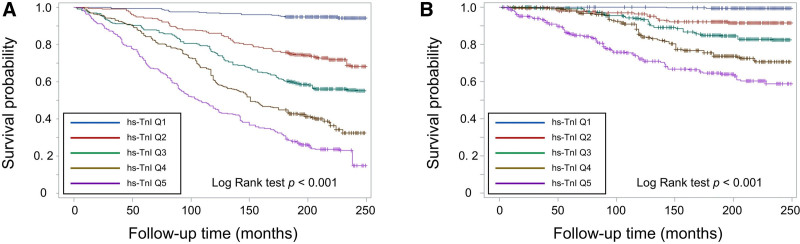
Survival curves for (A) all-cause and (B) cardiovascular mortality risk by quintiles of high-sensitivity troponin I (hs-TnI) levels. Q1 = Quintile 1 (0–1.2 ng/dL) (n = 256); Q2 = Quintile 2 (1.3–2.1 ng/dL) (n = 250); Q3 = Quintile 3 (2.2–3.4 ng/dL) (n = 263); Q4 = Quintile 4 (3.5–6.5 ng/dL) (n = 257); Q5 = Quintile 5 (6.6–862.8 ng/dL) (n = 261).

**Figure 2.**
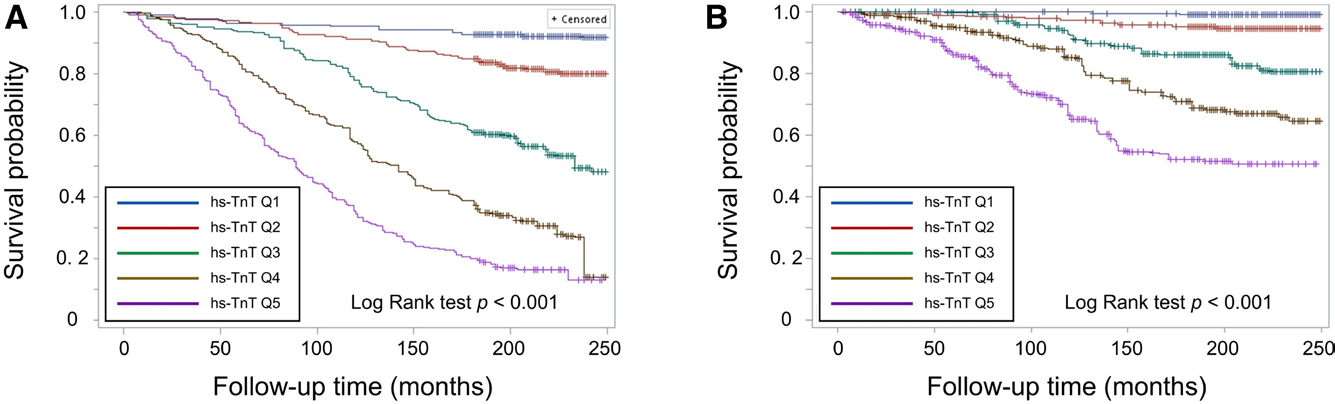
Survival curves for (A) all-cause and (B) cardiovascular mortality risk by quintiles of high-sensitivity troponin T (hs-TnT) levels. Q1 = Quintile 1 (0–4.07 ng/dL) (n = 256); Q2 = Quintile 2 (4.08–6.19 ng/dL) (n = 256); Q3 = Quintile 3 (6.20–9.78 ng/dL) (n = 256); Q4 = Quintile 4 (9.79–15.86 ng/dL) (n = 256); Q5 = Quintile 5 (15.87–214.4 ng/dL) (n = 255).

Over a median follow-up of 219 months (IQR: 199–233 months), a total of 601 participants died, including 185 from CVD, corresponding to a crude mortality rate of 28.4 per 10,000 person-months. Kaplan–Meier analysis showed progressively worse all-cause and CVD-related survival across increasing quintiles of hs-TnI (Fig. 1). In multivariable Cox models adjusting for age, sex, race/ethnicity, BMI, diabetes, hypertension, CVD, smoking status, family income-to-poverty ratio, hemoglobin, UACR, and eGFR, each one-quintile increase in hs-TnI was associated with a 26% higher risk of all-cause mortality (HR: 1.26, 95% CI: 1.13–1.41, *P*-trend < .001) and a 33% higher risk of CVD-related mortality (HR: 1.33, 95% CI: 1.04–1.71, *P*-trend < .05) (Table 3). Compared to those in the third quintile, participants in the highest quintile had a significantly greater risk of all-cause mortality (HR: 1.90, 95% CI: 1.31–2.76, *P* < .01) and CVD-related mortality (HR: 2.09, 95% CI: 1.07–4.09, *P* < .05). Conversely, those in the lowest quintile had a lower risk of all-cause mortality (HR: 0.51, 95% CI: 0.27–0.98, *P* < .05), though no significant association was observed with CVD-related mortality (Table 3). A similar pattern was observed for hs-TnT (Fig. 2, Table 3), where each one-quintile increase in hs-TnT was associated with a 56% higher risk of all-cause mortality (HR: 1.56, 95% CI: 1.37–1.77, *P* < .001) and a 64% higher risk of CVD-related mortality (HR: 1.64, 95% CI: 1.33–2.03, *P* < .001).

**Table 3:** All-cause and cardiovascular mortality risk by quintile of high-sensitivity troponin I (hs-TnI) and troponin T (hs-TnT) levels.

	All-cause mortality HR (95% CI)	Cardiovascular mortality HR (95% CI)
hs-TnI		
Quintile 1 (0–1.2 ng/dL) (n = 256)	0.51 (0.27–0.98)*	0.21 (0.04–1.08)
Quintile 2 (1.3–2.1 ng/dL) (n = 250)	1.19 (0.74–1.89)	1.25 (0.41–3.85)
Quintile 3 (2.2–3.4 ng/dL) (n = 263)	1	1
Quintile 4 (3.5–6.5 ng/dL) (n = 257)	1.16 (0.80–1.69)	1.02 (0.51–2.03)
Quintile 5 (6.6–862.8 ng/dL) (n = 261)	1.90 (1.31–2.76)**	2.09 (1.07–4.09)*
Trend	1.26 (1.13–1.41)***	1.33 (1.04–1.71)*
hs-TnT		
Quintile 1 (0–4.07 ng/dL) (n = 256)	0.54 (0.26–1.12)	0.05 (0.01–0.42)**
Quintile 2 (4.08–6.19 ng/dL) (n = 256)	0.64 (0.39–1.06)	0.82 (0.40–1.65)
Quintile 3 (6.20–9.78 ng/dL) (n = 256)	1	1
Quintile 4 (9.79–15.86 ng/dL) (n = 256)	1.43 (1.08–1.88)*	1.32 (0.77–2.26)
Quintile 5 (15.87–214.4 ng/dL) (n = 255)	2.59 (1.87–3.59)***	2.62 (1.61–4.27)***
Trend	1.56 (1.37–1.77)***	1.64 (1.33–2.03)***

Adjusted for age, sex, race/ethnicity, body mass index, diabetes, hypertension, cardiovascular disease, smoking status, hemoglobin, urinary albumin-creatinine ratio, estimated glomerular filtration rate, and family income-to-poverty ratio.

* *P* < .05.

** *P* < .01.

*** *P* < .001.

Findings were consistent for hs-TnI measured using the Ortho method, whereas no significant associations were observed for hs-TnI measured using the Siemens method (Table 4). In sensitivity analyses excluding individuals with self-reported CVD, the associations between hs-TnI and all-cause mortality remained robust. Each one-quintile increase in hs-TnI was associated with a 20% higher risk of all-cause mortality (HR: 1.20, 95% CI: 1.06–1.35, *P*-trend < .01) (Table 5). Compared to those in the third quintile, participants in the highest quintile had a significantly increased risk of all-cause mortality (HR: 1.81, 95% CI: 1.25–2.61, *P* < .01). However, the association between hs-TnI and CVD-related mortality was not statistically significant (Table 5). For hs-TnT, the associations remained significant even after excluding individuals with CVD. Each one-quintile increase was associated with a 45% higher risk of all-cause mortality (HR: 1.45, 95% CI: 1.26–1.67, *P* < .001) and a 47% higher risk of CVD-related mortality (HR: 1.47, 95% CI: 1.14–1.90, *P* < .01). Participants in the highest quintile had significantly increased risks of all-cause mortality (HR: 2.47, 95% CI: 1.68–3.65, *P* < .001) and CVD-related mortality (HR: 2.34, 95% CI: 1.35–4.05, *P* < .01) compared to those in the third quintile (Table 5).

**Table 4:** All-cause and cardiovascular mortality risk by quintile of high-sensitivity troponin I (hs-TnI) levels measured using Ortho Vitros 3600 and Siemens Centaur XP assays.

	All-cause mortality HR (95% CI)	Cardiovascular mortality HR (95% CI)
hs-TnI (Ortho)		
Quintile 1 (0–0.17 ng/L) (n = 256)	0.62 (0.33–1.15)	0.20 (0.05–0.77)*
Quintile 2 (0.18–0.64 ng/L) (n = 260)	1.07 (0.75–1.53)	0.91 (0.41–2.01)
Quintile 3 (0.65–1.37 ng/L) (n = 251)	1	1
Quintile 4 (1.38–3.08 ng/L) (n = 257)	1.10 (0.78–1.55)	0.77 (0.42–1.41)
Quintile 5 (1.39–819.9 ng/L) (n = 253)	2.00 (1.42–2.83)***	1.72 (0.89–3.32)
Trend	1.28 (1.15–1.42)***	1.34 (1.00–1.79)*
hs-TnI (Siemens)		
Quintile 1 (0–1.49 ng/L) (n = 251)	0.95 (0.56–1.63)	0.99 (0.29–3.31)
Quintile 2 (1.50–3.16 ng/L) (n = 248)	0.88 (0.56–1.37)	0.97 (0.51–1.84)
Quintile 3 (3.17–5.60 ng/L) (n = 250)	1	1
Quintile 4 (5.61–11.19 ng/L) (n = 249)	1.05 (0.78–1.42)	1.06 (0.65–1.73)
Quintile 5 (11.20–1028.45 ng/L) (n = 249)	1.26 (0.82–1.92)	1.45 (0.78–2.70)
Trend	1.10 (0.99–1.23)	1.13 (0.93–1.37)

Adjusted for age, sex, race/ethnicity, body mass index, diabetes, hypertension, cardiovascular disease, smoking status, hemoglobin, urinary albumin-creatinine ratio, estimated glomerular filtration rate, and family income-to-poverty ratio.

* *P* < .05.

*** *P* < .001.

**Table 5:** All-cause and cardiovascular mortality risk by quintile of high-sensitivity troponin I (hs-TnI) and troponin T (hs-TnT) levels excluding participants with preexisting cardiovascular disease.

	All-cause mortality HR (95% CI)	Cardiovascular mortality HR (95% CI)
hs-TnI		
Quintile 1 (0–1.2 ng/dL) (n = 193)	0.61 (0.30–1.23)	0.33 (0.06–1.86)
Quintile 2 (1.3–2.1 ng/dL) (n = 221)	1.17 (0.73–1.87)	1.37 (0.39–4.86)
Quintile 3 (2.2–3.4 ng/dL) (n = 226)	1	1
Quintile 4 (3.5–6.5 ng/dL) (n = 189)	0.97 (0.60–1.55)	1.13 (0.44–2.89)
Quintile 5 (6.6–862.8 ng/dL) (n = 171)	1.81 (1.25–2.61)*	2.05 (0.85–4.96)
Trend	1.20 (1.06–1.35)*	1.27 (0.90–1.78)
hs-TnT		
Quintile 1 (0–4.07 ng/dL) (n = 200)	0.68 (0.32–1.45)	0.08 (0.01–0.67)**
Quintile 2 (4.08–6.19 ng/dL) (n = 212)	0.77 (0.45–1.32)	1.05 (0.54–2.06)
Quintile 3 (6.20–9.78 ng/dL) (n = 222)	1	1
Quintile 4 (9.79–15.86 ng/dL) (n = 189)	1.47 (1.04–2.08)**	1.38 (0.77–2.48)
Quintile 5 (15.87–214.4 ng/dL) (n = 169)	2.47 (1.68–3.65)***	2.34 (1.35–4.05)*
Trend	1.45 (1.26–1.67)***	1.47 (1.14–1.90)*

Adjusted for age, sex, race/ethnicity, body mass index, diabetes, hypertension, smoking status, hemoglobin, urinary albumin-creatinine ratio, estimated glomerular filtration rate, and family income-to-poverty ratio.

* *P* < .01.

** *P* < .05.

*** *P* < .001.

To address potential reverse causation, a secondary analysis was conducted excluding participants who died within the first 2 years of follow-up. The results remained consistent with the primary findings. Each one-quintile increase in hs-TnI was associated with a 21% higher risk of all-cause mortality (HR: 1.21, 95% CI: 1.09–1.36, *P*-trend < .01) (Table 6). Participants in the highest quintile had a significantly increased risk of all-cause mortality compared to those in the third quintile (HR: 1.85, 95% CI: 1.27–2.69, *P* < .01), but the association with CVD-related mortality remained nonsignificant (Table 6). For hs-TnT, the associations remained robust. Each one-quintile increase in hs-TnT was associated with a 54% higher risk of all-cause mortality (HR: 1.54, 95% CI: 1.35–1.76, *P* < .001) and a 61% higher risk of CVD-related mortality (HR: 1.61, 95% CI: 1.27–2.03, *P* < .001). Participants in the highest quintile had significantly increased risks of all-cause mortality (HR: 2.71, 95% CI: 1.98–3.72, *P* < .001) and CVD-related mortality (HR: 2.37, 95% CI: 1.41–4.00, *P* < .001) compared to those in the third quintile (Table 6).

**Table 6:** All-cause and cardiovascular mortality risk by quintile of high-sensitivity troponin I (hs-TnI) and troponin T (hs-TnT) levels excluding participants who died within the first 2 years of follow-up.

	All-cause mortality HR (95% CI)	Cardiovascular mortality HR (95% CI)
hs-TnI		
Quintile 1 (0–1.2 ng/dL) (n = 256)	0.59 (0.31–1.12)	0.21 (0.04–1.13)
Quintile 2 (1.3–2.1 ng/dL) (n = 247)	1.31 (0.82–2.07)	1.19 (0.37–3.77)
Quintile 3 (2.2–3.4 ng/dL) (n = 256)	1	1
Quintile 4 (3.5–6.5 ng/dL) (n = 240)	1.19 (0.81–1.75)	1.00 (0.50–2.03)
Quintile 5 (6.6–862.8 ng/dL) (n = 235)	1.85 (1.27–2.69)**	1.74 (0.87–3.49)
Trend	1.21 (1.09–1.36)**	1.25 (0.98–1.61)
hs-TnT		
Quintile 1 (0–4.07 ng/dL) (n = 255)	0.62 (0.30–1.29)	0.06 (0.01–0.44)**
Quintile 2 (4.08–6.19 ng/dL) (n = 254)	0.70 (0.43–1.13)	0.74 (0.35–1.55)
Quintile 3 (6.20–9.78 ng/dL) (n = 249)	1	1
Quintile 4 (9.79–15.86 ng/dL) (n = 245)	1.49 (1.11–1.99)**	1.21 (0.71–2.06)
Quintile 5 (15.87–214.4 ng/dL) (n = 223)	2.71 (1.98–3.72)***	2.37 (1.41–4.00)***
Trend	1.54 (1.35–1.76)***	1.61 (1.27–2.03)***

Adjusted for age, sex, race/ethnicity, body mass index, diabetes, hypertension, cardiovascular disease, smoking status, hemoglobin, urinary albumin-creatinine ratio, estimated glomerular filtration rate, and family income-to-poverty ratio.

** *P* < .01.

*** *P* < .001.

## 4. Discussion

In this study of individuals with early kidney disease, elevated hs-TnI and hs-TnT levels were independently associated with increased risks of all-cause and cardiovascular mortality. These associations remained consistent after excluding individuals with preexisting CVD and those who died within the first 2 years of follow-up, suggesting that subclinical elevations in cardiac troponins reflect early pathophysiological processes that precede overt cardiovascular events. Participants with obesity and higher blood pressure were more likely to have elevated hs-TnI, while those with diabetes, hypertension, CVD, lower hemoglobin, and higher UACR were more likely to have elevated hs-TnT. These findings suggest that hs-Tn may be useful markers for cardiovascular risk stratification.

Cardiac troponins are primarily intracellular, with over 90% bound within the sarcomere and the remainder free in the cytoplasm.^[20]^ While their release into circulation is traditionally linked to myocardial necrosis, emerging evidence suggests multiple nonnecrotic mechanisms, including apoptosis, increased membrane permeability, proteolytic degradation, and release from membranous blebs.^[20]^ In individuals with early kidney disease, several interconnected processes contribute to subclinical myocardial injury.^[21–25]^ Hemodynamic overload from hypertension, arterial stiffness, and volume shifts promotes left ventricular hypertrophy and myocardial strain.^[21–25]^ Endothelial dysfunction and microvascular disease, both prevalent in albuminuria, may impair coronary perfusion,^[24,25]^ leading to silent myocardial ischemia and chronic myocardial stress. Additionally, low-grade systemic inflammation and oxidative stress, hallmarks of kidney dysfunction, contribute to cardiomyocyte apoptosis, fibrosis, and myocardial remodeling.^[2]^ These pathophysiological changes not only cause continuous troponin release but also set the stage for heart failure, arrhythmias, and sudden cardiac death, major contributors to elevated cardiovascular and all-cause mortality in this population.^[26]^ Thus, elevated hs-Tn levels in individuals with early kidney disease likely reflects cumulative subclinical myocardial injury, serving as an early and sensitive biomarker of increased risk for future fatal cardiovascular events.

Our findings are consistent with previous studies showing that elevated hs-Tn levels are predictive of all-cause and cardiovascular mortality in the general population,^[27]^ individuals with CKD,^[10–12]^ and those with established CVD.^[9]^ Importantly, even low-level elevations in hs-Tn have been shown to carry prognostic significance,^[6–9,28]^ reinforcing the utility of these biomarkers beyond traditional cardiovascular risk factors. While prior research has primarily focused on patients with advanced CKD^[10–12]^ or established CVD,^[9]^ our study extends these observations to individuals with early-stage kidney disease, highlighting that subclinical elevations in hs-TnI and hs-TnT remain robust predictors of long-term mortality risk in this population. Notably, in our multivariable models, elevated hs-Tn remained significantly associated with mortality after adjustment for conventional risk markers, suggesting that hs-Tn assays may provide additional predictive value beyond traditional markers such as eGFR and albuminuria. These results support the potential role of hs-Tn in enhancing cardiovascular risk stratification among individuals with early kidney disease and underscore the need for future research to determine whether integrating hs-Tn testing into routine clinical assessments could improve risk prediction and guide preventive strategies.

Our findings align with previous research suggesting that hs-TnT may have a stronger association with mortality than hs-TnI, particularly in individuals with kidney dysfunction.^[11,12]^ This difference could be attributed to its partial renal clearance,^[29–32]^ which may result in higher circulating levels in individuals with impaired kidney function, potentially making it a more sensitive indicator of subclinical cardiovascular stress. Additionally, hs-TnT has been more closely linked to chronic myocardial injury, fibrosis, and left ventricular remodeling,^[33]^ whereas hs-TnI is thought to be more specific for acute myocardial necrosis.^[23]^ Given that early kidney disease is often associated with chronic cardiac remodeling rather than acute ischemic events,^[34]^ hs-TnT may provide additional prognostic value in this population. However, further studies are needed to determine whether these differences have clinical implications for risk stratification and management.

A major strength of this study is the use of a nationally representative cohort with long-term follow-up, allowing for a robust assessment of the association between hs-Tn levels and mortality risk in individuals with early kidney disease. The large sample size and comprehensive adjustment for confounders enhance the validity of our findings. Additionally, the inclusion of both hs-TnI and hs-TnT provides insights into their comparative prognostic value, with hs-TnT potentially being more predictive of long-term outcomes in individuals with kidney dysfunction. Furthermore, the consistency of our results across sensitivity analyses, including the exclusion of individuals with preexisting CVD and early deaths, supports the robustness of our conclusions and minimizes the risk of reverse causation.

However, this study has several limitations. First, hs-Tn levels were measured at a single time point, preventing the assessment of longitudinal changes or whether dynamic fluctuations provide additional prognostic value. Second, although we adjusted for major confounders, residual confounding from unmeasured factors, such as cardiac structural abnormalities, subclinical inflammation, or genetic predispositions, cannot be ruled out. Third, while albuminuria was used to define early kidney disease, other markers of kidney function, such as cystatin C, were not included, which may have provided a more comprehensive assessment of kidney health. Lastly, as an observational study, causality cannot be established. Despite these limitations, our findings provide insights into risk stratification for individuals with early kidney disease. The observed association between elevated hs-Tn levels and mortality suggests that hs-Tn may serve as an early marker of myocardial injury, even in the absence of overt clinical symptoms.

Our findings have important implications for clinical practice and public health. Individuals with albuminuria but preserved eGFR are often under-recognized as being at high cardiovascular risk. The strong association between elevated hs-Tn levels and mortality suggests that hs-Tn may serve as a valuable biomarker for early identification of individuals who could benefit from intensified cardiovascular risk management. Although no current interventions specifically target elevated hs-Tn levels, therapies that reduce cardiovascular stress, such as sodium-glucose cotransporter-2 inhibitors, renin–angiotensin–aldosterone system blockers, and statins, may help mitigate underlying myocardial injury and improve clinical outcomes. Moreover, the observed associations between hs-Tn, obesity, and hypertension underscore the importance of lifestyle interventions, such as blood pressure control, weight reduction, and metabolic optimization, in potentially improving long-term outcomes. Future research should evaluate whether serial monitoring of hs-Tn levels or targeted interventions based on these biomarkers can improve clinical outcomes. Given the differential prognostic patterns observed between hs-TnI and hs-TnT, further research is warranted to determine whether hs-TnT provides incremental value for risk stratification and clinical decision-making in individuals with early kidney disease.

In conclusion, among individuals with early kidney disease, elevated hs-TnI and hs-TnT levels were independently associated with increased risks of all-cause and cardiovascular mortality. These findings suggest that subclinical myocardial injury, as indicated by elevated hs-Tn, may be an early marker of cardiovascular risk. Since traditional risk models may not fully capture cardiovascular risk in individuals with albuminuria, incorporating hs-Tn measurement into risk assessment could provide additional prognostic value by identifying high-risk individuals who may benefit from earlier interventions, potentially before overt symptoms or clinical events occur. Further research is needed to determine the clinical utility of hs-Tn for ongoing monitoring and to evaluate whether its incorporation into routine clinical practice can improve outcomes. If validated in prospective studies, these findings could inform future risk stratification guidelines and public health strategies aimed at reducing cardiovascular mortality in the growing population with early kidney disease.

## Author contributions

**Conceptualization:** Ming-Yan Jiang.

**Data curation:** Ming-Yan Jiang.

**Formal analysis:** Ming-Yan Jiang.

**Investigation:** Wen-Shiann Wu.

**Methodology:** Ming-Yan Jiang, Wen-Shiann Wu.

**Software:** Ming-Yan Jiang.

**Writing – original draft:** Ming-Yan Jiang.

**Writing – review & editing:** Ming-Yan Jiang, Wen-Shiann Wu.

## Supplementary Material


