# Supplementary material for: Association between high-sensitivity troponin and mortality risk in individuals with early kidney disease: A population-based cohort study
Source: Medicine (Baltimore). 2025 Jun 27;104(26):e43014. doi: 10.1097/MD.0000000000043014 (PMC12212771; doi:10.1097/MD.0000000000043014)
Supplement: Supplementary file 1 [file medi-104-e43014-s001.docx]

**Association between high-sensitivity troponin and mortality risk in individuals with early kidney disease: a population-based cohort study**

Ming-Yan Jiang, MD, MSc ^1,2*^, Wen-Shiann Wu, MD ^3*^

^1^ Renal Division, Department of Internal Medicine, Chi Mei Medical Center, Tainan, Taiwan

^2^ Department of Pharmacy, Chia Nan University of Pharmacy & Science, Tainan, Taiwan

^3^ Cardiovascular Division, Department of Internal Medicine, Chi Mei Medical Center, Tainan, Taiwan

Supplemental Table S1. Factors associated with high-sensitivity troponin I levels (log transformed) by multiple linear regression

| Parameter | ß coefficient | Standard error | 95% CI lower bound | 95% CI upper bound | *p* value |
| --- | --- | --- | --- | --- | --- |
| Intercept | -0.2754 | 0.7017 | -1.6896 | 1.1387 | 0.70 |
| **Age** (every 10 years old) | 0.1041 | 0.0083 | 0.0873 | 0.1209 | < 0.001 |
| **Male** (vs. female) | 0.1892 | 0.0275 | 0.1337 | 0.2446 | < 0.001 |
| **Race/ethnicity** |  |  |  |  |  |
| White | ref |  |  |  |  |
| Black | 0.1218 | 0.0378 | 0.0456 | 0.1981 | < 0.01 |
| Hispanic | -0.0047 | 0.0309 | -0.0669 | 0.0575 | 0.88 |
| Other | 0.0907 | 0.0870 | -0.0846 | 0.2659 | 0.30 |
| **Family income-to-poverty ratio** | -0.0159 | 0.0071 | -0.0302 | -0.0015 | < 0.05 |
| **Waist circumference** (every 10 cm) | 0.0192 | 0.0065 | 0.0061 | 0.0323 | < 0.01 |
| **Smoking status** |  |  |  |  |  |
| Never | ref |  |  |  |  |
| Former | -0.0136 | 0.0246 | -0.0631 | 0.0359 | 0.58 |
| Current | 0.0003 | 0.0338 | -0.0678 | 0.0685 | 0.99 |
| **Diabetes** | -0.0303 | 0.0309 | -0.0926 | 0.0320 | 0.33 |
| **Hypertension** | 0.1218 | 0.0254 | 0.0705 | 0.1731 | < 0.001 |
| **Cardiovascular disease** | 0.2001 | 0.0459 | 0.1077 | 0.2926 | < 0.001 |
| **UACR** ^#^ | 0.0556 | 0.0316 | -0.0081 | 0.1194 | 0.09 |
| **eGFR** ^#^ | -0.1446 | 0.1621 | -0.4713 | 0.1821 | 0.38 |
| **Hemoglobin** ^#^ | 0.0062 | 0.4979 | -0.9973 | 1.0097 | 0.99 |

Abbreviation: UACR: urinary albumin-creatinine ratio; eGFR: estimated glomerular filtration rate.

^#^: Log transformed
